# Assessing the Effectiveness of a Structured Teaching-Learning Module in Improving Knowledge and Skills in Writing Injury Reports Among the Undergraduate Medical Students of a Tertiary Care Center in India

**DOI:** 10.7759/cureus.75824

**Published:** 2024-12-16

**Authors:** Kaustav Bairagi, Vijay Kumar, Samarendra Barman, Subhrajeet Chakraborty

**Affiliations:** 1 Department of Forensic Medicine and Toxicology, All India Institute of Medical Sciences-Guwahati, Guwahati, IND; 2 Department of Forensic Medicine and Toxicology, Shri Ram Murti Smarak Institute of Medical Sciences-Bareilly, Bareilly, IND; 3 Department of Community and Family Medicine, All India Institute of Medical Sciences-Guwahati, Guwahati, IND

**Keywords:** injury report writing, knowledge and skill, medical students, performance, structured teaching-learning module

## Abstract

Background

The ability to write accurate and comprehensive injury reports is a crucial skill for medical professionals, particularly those working in emergency medicine and trauma care. A structured teaching-learning (TL) module can enhance the knowledge and skills of undergraduate medical students in this area.

Aim

The study aims to assess students’ performance in cognitive, psychomotor, and communication domains of injury report writing before and after implementing the proposed structured TL module and compare the findings to evaluate the efficacy between the existing TL module and the proposed module.

Methods

A total of 50 undergraduate Bachelor of Medicine, Bachelor of Surgery (MBBS) students of the tertiary care center consented to participate in the study. A total of 41 students participated throughout the entire study duration and were considered for data analysis. The performance of the students was evaluated through assessment and a structured grading description was utilized for distribution of marks. Data were collected, compiled, and analyzed with the help of IBM SPSS Statistics for Windows, Version 16 (Released 2007; IBM Corp., Armonk, New York, United States).

Results

The percentage of students scoring higher than 60% marks in all three domains after the implementation of the proposed module in both immediate post-implementation assessment and two months after post-implementation assessment in comparison to pre-implementation assessment has been found to be statistically significant (p-value < 0.01).

Conclusion

The study observed improvement of performance in all three domains of the competency after implementation of the structured TL module which can be attributed to the introduction of more interactive sessions both in the form of small-group learning and bedside learning in the proposed module.

## Introduction

Injury report writing is a critical skill that Indian medical graduates must possess. It plays a key role in the investigation and documentation of injuries in medicolegal cases as well as in the legal proceedings that may follow. By mastering this skill, medical graduates can ensure that their reports are informative, accurate, and comprehensive, ultimately contributing to the delivery of justice and the provision of quality medicolegal care.

Various research studies have observed a significant lack of knowledge and skills among medical graduates to write proper medicolegal reports in professional practice [1,2]. Incorrect or incomplete medicolegal reports may trigger a pause or delay in legal proceedings, and patients' rights could be violated. The authors have noted that the lack of standardized and structured teaching-learning (TL) modules for certain competencies of the medical curriculum may be one of the important reasons for such deficiency in knowledge and skills among medical professionals.

The conventional TL module, typically employed in many Indian medical colleges, for the development of knowledge and skills in writing injury reports, consists of a one-hour didactic lecture followed by two hours of practical sessions. Practical sessions may focus on rote learning rather than engaging students in real-life scenarios and may not be sufficient for the optimum development of knowledge and skills. Injury report writing often requires critical thinking, analysis of evidence, and clear communication, which might not be adequately addressed in a short unstructured practical session without real-world application.

This present study was done to assess the impact of implementing a modified structured TL module in improving the performance of cognitive, psychomotor, and communication domains of injury report writing skills among the study participants. The proposed module focuses more on small-group discussion and bedside learning during the practical session to measure the improvement of performance in the post-implementation assessment. 

## Materials and methods

The study was carried out among the MBBS students of Forensic Medicine and Toxicology in a tertiary care hospital in India for a period of one year from October 2021 to September 2022. This study aims to assess students' performance in cognitive, psychomotor, and communication domains of injury report writing before and after the implementation of the proposed structured TL module and compare the findings to evaluate the efficacy between the existing conventional TL module and the proposed module.

All students who participated in the study had basic knowledge and skills in injury report writing owing to prior exposure to the conventional TL module. The conventional TL module was comprised of one hour of didactic theory class and two hours of practical sessions in the demonstration room. The proposed module has one hour of didactic theory class, one hour of small-group discussion with one facilitator in each group, and one hour of bedside teaching of injury interpretation on live patients in clinics. The proposed module was implemented among the study participants on the same day to prevent bias. The total duration allotted for the proposed module was the same as that of the existing module. The content of learning and report format used were the same in both modules. There was an assessment done on the study participants before the implementation of the proposed module to measure their performance. Multiple choice questions (MCQs), short-answer questions (SAQs), and objective structured practical examination (OSPE) were used as assessment tools. Questionnaires were validated by external subject experts from other institutes. The study participants were assessed next day after the implementation of the proposed module to measure post-implementation performance. Immediate post-implementation assessment was taken based on the same questionnaires as that of the pre-implementation assessment. Two months after the implementation of the proposed module, a reassessment was conducted among the study participants without any prior intimation. In that reassessment, the questionnaires for MCQs and SAQs were phrased in different wording but expressed the same meaning and intent as that of the pre-implementation questionnaires. The objective of this phase of assessment was to evaluate the retention of knowledge and skills in injury report writing long after the implementation of the proposed TL module. Feedback was taken from the study participants about the proposed TL module and assessment methods. 

Ethical permission was obtained from the Institute Ethics Committee (IEC) before the commencement of the study. The participation of students was voluntary. The students were given explanations about the study, and informed consent was obtained from them. At the end of the study, the significance of the difference in performance was assessed before and after the implementation of the proposed structured TL module.

The study also evaluates the performance of the participants in relation to all three domains two months after the implementation of the novel module to assess the retention of knowledge and skills. Evaluation of the effectiveness of the structured TL module in comparison to the existing module is done based on the performance metrics. A structured grading description (Table 1) was utilized against the distribution of marks [3]. Marks scored above 60% is considered good and taken as a benchmark to see the improvement. Data were collected, compiled, and analyzed using IBM SPSS Statistics for Windows, Version 16 (Released 2007; IBM Corp., Armonk, New York, United States). 

**Table 1:** Grading description against the distribution of marks in percentage %

Percentage of marks	Grading description
1-20	Poor
21-40	Fair
41-60	Average
61-80	Good
81-100	Excellent

## Results

A total of 50 students from the second-year professional MBBS batch consented to participate in the study. Only 41 students (n = 41) attended the entire period of the study and assessment and hence were considered for data analysis. On day 1, a pre-implementation assessment was taken based on the expert-validated MCQs, SAQs, and OSPE. On day 2, the proposed TL module was implemented. On day 3, the participants were assessed again (post-implementation assessment) without prior intimation about the assessment. A reassessment was conducted two months after the implementation of the module.

During the pre-implementation assessment (Table 2), 83% (n = 41) of the students scored less than or equal to 60% in the cognitive domain. Only about 5% of the students scored above 80% marks. In the psychomotor domain, 19.4% (n = 41) of the students scored more than 60%. The majority of the students (56%; n = 41) scored 40% or less in the psychomotor domain. In the communication skill domain, about 85% (n = 41) of the students scored 60% or less. In that category, 4.8% of students scored 20% or less. It is to be noted that all the students who participated in the pre-implementation assessment already had gone through the conventional TL module on injury report writing. 

**Table 2:** Pre-implementation assessment performance represented in number n and percentage %

Scoring in the cognitive domain	
Distribution of marks in %	Number & percentage of students (n = 41)
1-20	00 (0)
21-40	22 (53.7%)
41-60	12 (29.2%)
61-80	05 (12.3%)
81-100	02 (4.8%)

Scoring in the psychomotor domain	
Distribution of marks in %	Number & percentage of students (n = 41)
1-20	00 (00%)
21-40	23 (56.0%)
41-60	10 (24.5%)
61-80	07 (17.0%)
81-100	01 (2.5%)

Scoring in communication skills	
Distribution of marks in %	Number & percentage of students (n = 41)
1-20	02 (4.8%)
21-40	19 (46.5%)
41-60	14 (34.1%)
61-80	05 (12.2%)
81-100	01 (2.4%)

During the immediate post-implementation assessment (Table 3), 44% (n = 41) of the participants scored in the range of 61%-80% in the cognitive domain. About 70% (n = 41) of the students scored more than 60% marks in the assessment. In the psychomotor domain, 70% (n = 41) of the students were able to score more than 60% marks, whereas 39% (n = 41) of the students scored in the range of 81%-100%. In the affective domain, 51% (n = 41) of the students scored more than 80% marks, and about 78% (n = 41) of the students scored more than 60%.

**Table 3:** Immediate post-implementation assessment performance represented in number n and percentage %

Scoring in the cognitive domain	
Distribution of marks in %	Number & percentage of students (n = 41)
1-20	00 (00%)
21-40	03 (7.3%)
41-60	09 (21.9%)
61-80	18 (43.9%)
81-100	11 (26.9%)

Scoring in the psychomotor domain	
Distribution of marks in %	Number & percentage of students (n = 41)
1-20	00 (00%)
21-40	04 (9.7%)
41-60	08 (19.6%)
61-80	13 (31.7%)
81-100	16 (39.0%)

Scoring in communication skills	
Distribution of marks in %	Number & percentage of students (n = 41)
1-20	00 (00%)
21-40	01 (2.4%)
41-60	08 (19.6%)
61-80	11 (26.8%)
81-100	21 (51.2%)

In the reassessment conducted two months after implementation (Table 4), 63% (n = 41) of the students scored more than 60% marks in the cognitive domain. This was 70% in the immediate post-implementation assessment and about 17% in the pre-implementation assessment. In the psychomotor domain, about 63% (n = 41) of the students scored more than 60% marks in that late assessment. This was 70% in the immediate post-implementation assessment and 19% in the pre-implementation assessment. In the communication domain, 73% (n = 41) of the students scored more than 60% marks which was 78% in the immediate post-implementation assessment and about 15% in the pre-implementation assessment. 

**Table 4:** Two months post-implementation assessment performance represented in number n and percentage %

Scoring in the cognitive domain	
Distribution of marks in %	Number & percentage of students (n = 41)
1-20	00 (00%)
21-40	05 (12.2%)
41-60	10 (24.4%)
61-80	16 (39.0%)
81-100	10 (24.4%)

Scoring in the psychomotor domain	
Distribution of marks in %	Number & percentage of students (n = 41)
1-20	00 (00%)
21-40	06 (14.6%)
41-60	09 (21.9%)
61-80	13 (31.8%)
81-100	13 (31.7%)

Scoring in communication skills	
Distribution of marks in %	Number & percentage of students (n = 41)
1-20	00 (00%)
21-40	03 (7.3%)
41-60	08 (19.5%)
61-80	13 (31.7%)
81-100	17 (41.5%)

## Discussion

Injury report writing is a core competency as per the National Medical Commission (NMC) curriculum and a must to learn for all undergraduate medical students in India [4]. Studies have reported a significant lack of knowledge and skills among medical graduates to write proper medicolegal reports in professional practice [1,2]. The conventional TL module, comprised of a total of three hours of learning with one hour of didactic theory lecture and two hours of practical session, may not be effective in inculcating adequate knowledge and skills among the students. The present study has proposed a structured TL module comprising one hour of theory lecture, one hour of small-group discussion, and one hour of bedside teaching for optimal improvement of cognitive, psychomotor, and communication skills related to injury report writing. In the proposed module, the theory lecture has been kept the same as that of the existing module, but the two hours of the practical session of the conventional module has been divided into one hour of interactive learning in a small group setting under a facilitator and one hour of bedside teaching on live patients in clinics. 

**Figure 1:**
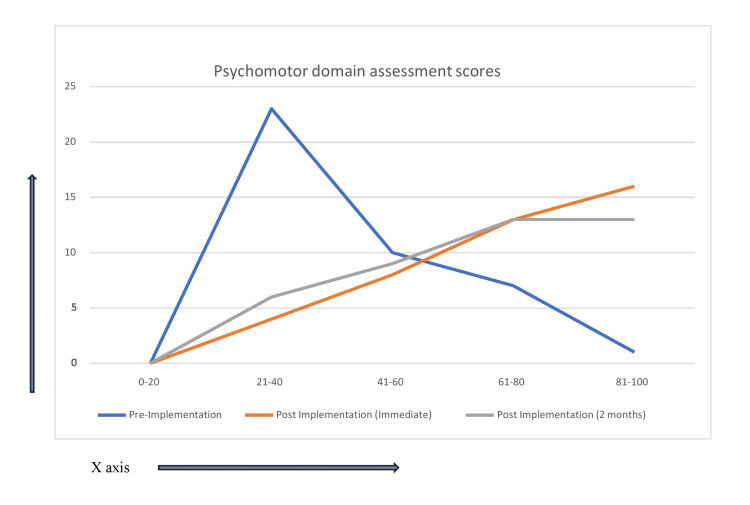
Performance in the psychomotor domain in all three assessments represented as line graph (x-axis = percentage % of marks and y-axis = number of students n)

**Figure 2:**
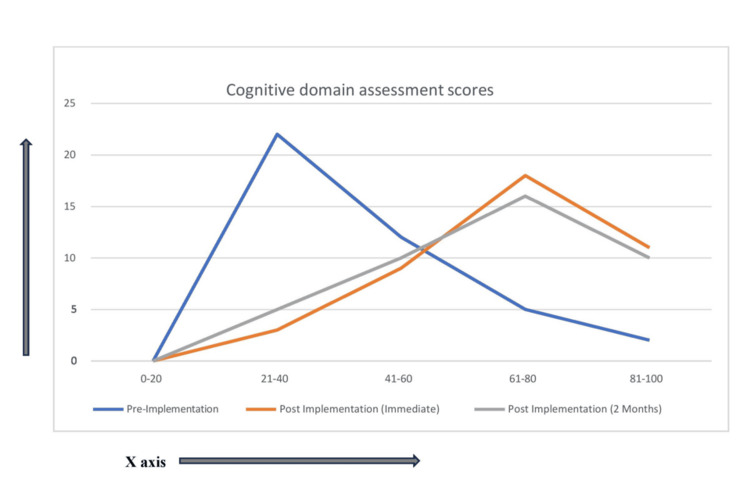
Performance in the cognitive domain in all three assessments represented as line graph (x-axis = percentage % of marks and y-axis = number of students n)

**Figure 3:**
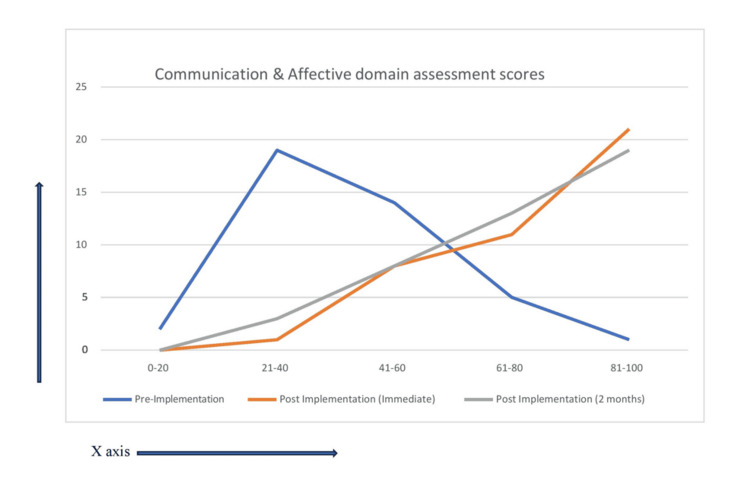
Performance in the communication domain in all three assessments represented as line graph (x-axis = percentage % of marks and y-axis = number of students n)

The study finding shows that students' performance was better in all three domains after the implementation of the proposed TL module (Figures 1-3). The percentage of students scoring higher than 60% marks in all three domains after implementation of the module in both immediate post-implementation assessment and two months post-implementation assessment is statistically significant in comparison to pre-implementation assessment (p-value < 0.01) (Tables 5-6). Perhaps it's because of the introduction of more interactive sessions both in the form of small group learning and bedside learning. Small groups encourage active participation from all members, as the smaller setting often makes individuals feel more comfortable sharing their thoughts and ideas. Besides, this setting also helps individuals practice and refine their verbal and non-verbal communication skills [5]. Bedside learning, which refers to the education and training that occurs in a clinical setting as opposed to a classroom, is another vital component of medical education. Bedside learning allows students to apply theoretical knowledge in real clinical scenarios, thereby enhancing understanding and retention. It bridges the gap between theoretical knowledge and its application, helping to produce competent, confident, and compassionate healthcare providers [6]. In a study by Nair et al, learners were found to believe that bedside teaching is a "valuable way to develop professional skills." More than 90% of the learners believed that bedside teaching is effective for learning communication, history-taking, and physical examination skills [7]. In another study, it is evident from the difference between pre- and post-test scores that clinical posting has increased the learning of medicolegal procedures in Forensic Medicine and Toxicology [8].

**Table 5:** Comparison of students' performance in assessments before and after implementation of the proposed TL module. Data represented in number n, percentage %, and p-value TL: teaching-learning

		<60%	>60%	(χ2 ; p-value)
Cognitive domain	Pre-implementation	34	7	23.9; <0.01
	Post-implementation (immediate)	12	29	
Psychomotor domain	Pre-implementation	33	8	21.7; <0.01
	Post-implementation (immediate)	12	29	
Affective domain	Pre-implementation	35	6	33.15; <0.01
	Post-implementation (immediate)	9	32	

**Table 6:** Comparison of students' performance before implantation and two months after implementation of the proposed TL module. Data represented in number n, percentage %, and p-value TL: teaching-learning

		<60%	>60%	(χ2 ; p-value)
Cognitive domain	Pre-implementation	34	7	18.3; <0.01
	Post-implementation (2 months post-implementation)	15	26	
Psychomotor domain	Pre-implementation	33	8	16.2; <0.01
	Post-implementation (2 months post-implementation)	15	26	
Affective domain	Pre-implementation	35	6	28.52; <0.01
	Post-implementation (2 months post-implementation)	11	30	

The findings noted in Table 4 and Table 6 reveal that even after an elapse of two months following the implementation of the proposed module, the study participants performed better in all three domains in comparison to the pre-implementation assessment. This shows that the study participants could retain the knowledge and skills of writing injury reports effectively for a longer duration because of better learning and understanding during the implementation of the proposed module. This improvement can be attributed to small-group discussions and bedside learning incorporated in the proposed module. Several research studies have observed that acquisition and retention of knowledge and skills related to a particular competency is found to be better if small-group learning and bedside teaching at clinics are incorporated into the TL module [9,10]. 

However, the effectiveness of the proposed module can be influenced by several factors including the quality of instruction, the clinical environment, and institutional support for education. The other major drawback that could be noted in the proposed module is that more manpower has to be allotted for implementation because of the need for facilitators in small-group learning. The role of the facilitator is to "facilitate" the learning: lead the discussion, ask open-ended questions, guide the process, and ensure active participation from students [11]. This can be a major challenge in institutes with manpower constraints, more so for a large batch which may consist of students with varied learning styles, backgrounds, and levels of understanding. The utilization of trained tutors or resident doctors as a facilitator can solve these problems to some extent. Designing faculty development training for tutors or resident doctors to improve the effective delivery of knowledge and skill and for adopting and effectively utilizing novel tools for learning and assessment cannot be overemphasized [12].

The authors have identified a few limitations of the study which include a small sample size, lack of a control group, confounding variables, and students' motivation and engagement. With a limited number of participants, a small sample may impact the statistical power of the study. Without a control group (students who do not receive the structured TL module), it becomes difficult to conclusively determine if the improvements are solely due to the module or other external factors. Factors such as prior experience in writing reports, exposure to real-life injury cases, or previous training, might influence the students' performance. These variables can affect the outcomes and create difficulty in attributing the observed improvements directly to the module. Students who are less motivated or engaged in the learning process might show less improvement, which could skew the results. By recognizing these limitations, authors can refine future studies or interpret their findings with a degree of caution.

## Conclusions

The science of medical education is evolving rapidly. Students’ performance in a core competency depends on both the quality of learning content and methodologies and the quality of faculties delivering the content. By creating a structured TL module that emphasizes small-group learning and bedside learning, educators can develop an engaging and effective environment that fosters knowledge and skills in the recipients. The goal is to ensure that all medical graduates can competently enhance their knowledge and skills in injury report writing, ultimately leading to better patient care and legal outcomes. 
